# HLA Alleles B^*^53:01 and C^*^06:02 Are Associated With Higher Risk of *P. falciparum* Parasitemia in a Cohort in Uganda

**DOI:** 10.3389/fimmu.2021.650028

**Published:** 2021-03-19

**Authors:** Jean C. Digitale, Perri C. Callaway, Maureen Martin, George Nelson, Mathias Viard, John Rek, Emmanuel Arinaitwe, Grant Dorsey, Moses Kamya, Mary Carrington, Isabel Rodriguez-Barraquer, Margaret E. Feeney

**Affiliations:** ^1^Department of Medicine, University of California, San Francisco, San Francisco, CA, United States; ^2^Department of Epidemiology and Biostatistics, University of California, San Francisco, San Francisco, CA, United States; ^3^Infectious Disease and Immunity Graduate Group, University of California, Berkeley, Berkeley, CA, United States; ^4^Basic Science Program, Frederick National Laboratory for Cancer Research in the Laboratory of Integrative Cancer Immunology, National Cancer Institute, Bethesda, MD, United States; ^5^Advanced Biomedical Computational Science, Frederick National Laboratory for Cancer Research, Frederick, MD, United States; ^6^Infectious Diseases Research Collaboration, Kampala, Uganda; ^7^London School of Hygiene and Tropical Medicine, London, United Kingdom; ^8^Department of Medicine, Makerere University, Kampala, Uganda; ^9^Ragon Institute of MGH MIT and Harvard, Cambridge, MA, United States; ^10^Department of Pediatrics, University of California, San Francisco, San Francisco, CA, United States

**Keywords:** HLA, MHC, malaria, *Plasmodium falciparum*, immunogenetics

## Abstract

Variation within the HLA locus been shown to play an important role in the susceptibility to and outcomes of numerous infections, but its influence on immunity to *P. falciparum* malaria is unclear. Increasing evidence indicates that acquired immunity to *P. falciparum* is mediated in part by the cellular immune response, including NK cells, CD4 and CD8 T cells, and semi-invariant γδ T cells. HLA molecules expressed by these lymphocytes influence the epitopes recognized by *P. falciparum*-specific T cells, and class I HLA molecules also serve as ligands for inhibitory receptors including KIR. Here we assessed the relationship of HLA class I and II alleles to the risk of *P. falciparum* infection and symptomatic malaria in a cohort of 892 Ugandan children and adults followed prospectively via both active and passive surveillance. We identified two HLA class I alleles, HLA-B^*^53:01 and HLA-C^*^06:02, that were associated with a higher prevalence of *P. falciparum* infection. Notably, no class I or II HLA alleles were found to be associated with protection from *P. falciparum* parasitemia or symptomatic malaria. These findings suggest that class I HLA plays a role in the ability to restrict parasitemia, supporting an essential role for the cellular immune response in *P. falciparum* immunity. Our findings underscore the need for better tools to enable mechanistic studies of the T cell response to *P. falciparum* at the epitope level and suggest that further study of the role of HLA in regulating pre-erythrocytic stages of the *P. falciparum* life cycle is warranted.

## Introduction

*Plasmodium falciparum (P. falciparum)* malaria remains a leading cause of morbidity and mortality in sub-Saharan Africa, particularly in children. As a result of thousands of years of co-evolution, malaria has exerted strong selective pressure on the human genome. Prior studies have identified numerous host genetic variants that influence susceptibility to malaria (1), most of which impact the structure and function of erythrocytes. However, only a small proportion of the genetic resistance to malaria is explained by these known variants (2) and surprisingly few are implicated in the host immune response.

Human leukocyte antigens (HLA) play an important role in the cellular immune response to many infectious diseases, both by presenting antigen to T cells and by acting as ligands for immune receptors, including killer immunoglobulin-like receptors (KIR). HLA antigens are encoded in the most variable region in the human genome, the major histocompatibility complex, enabling the presentation of diverse pathogen-derived peptides. Many studies have observed associations between HLA class I and class II alleles and susceptibility to or outcomes of numerous viral, bacterial, and parasitic infections (3). It is increasingly appreciated that the cellular immune response (including CD8 and CD4 T cells, NK cells and γδ T cells) plays an important role in immunity to *P. falciparum* (4, 5), particularly at the pre-erythrocytic stages of infection when the parasite resides within HLA-bearing hepatocytes and the multiplicity of infection is lowest. Despite this, the influence of HLA on susceptibility to malaria has not been rigorously assessed in the context of cohort studies that incorporate careful longitudinal measurement of both *P. falciparum* parasitemia and clinical malaria.

To date, studies examining the relationship of HLA class I and II alleles with malaria susceptibility have yielded inconsistent results. Several studies have reported alleles that were associated with malaria susceptibility or protection (6–11), but few of these associations have been replicated across cohorts. Most HLA association studies that have been performed to date utilized a case-control study design, comparing severe malaria cases to non-severe controls (6, 8–11). Relatively few have examined susceptibility to non-severe malaria or asymptomatic parasitemia, despite the high likelihood that the immunologic mechanisms required for the restriction of parasitemia differ from those underlying severe malaria. A recent review called for more studies of longitudinally sampled asymptomatic infections to better understand molecular drivers of such infections (12). An additional challenge in field-based studies is that exposure to *P. falciparum*-infected mosquitoes can be quite heterogeneous, even within a small geographic area, which may complicate the identification of protective alleles.

Here, we assessed the effect of HLA class I and II alleles on measures of *P. falciparum* parasitemia and non-severe malarial disease in a large prospective cohort study of Ugandan children and adults. Our analyses incorporated household-level entomological data to control for heterogeneity in environmental exposure to infected mosquitos. Our goal was to identify HLA alleles that associate with protection from *P. falciparum* infection and/or clinical malaria in order to better understand the cellular mechanisms underlying antimalarial immunity.

## Methods

### Clinical and Parasitological Outcomes

Data were collected from a cohort study conducted in parallel at three sites in Uganda with a range of malaria transmission intensities which was part of the East Africa International Center of Excellence for Malaria Research. Nagongera, in Tororo district, is a rural area in southeastern Uganda with high transmission. Kihihi, in Kanungu district, is a rural area in southwestern Uganda with moderate transmission. Walukuba, in Jinja district, is a peri-urban area near Lake Victoria with relatively low transmission. Approximately 100 households were selected at each site, and all children aged 6 months to 10 years and one adult caregiver were offered enrolment. Participants were enrolled between August and October 2011 and followed through June 2016. In this analysis, visits after December 31, 2014 from Tororo were excluded because transmission changed dramatically at this site after an indoor residual spraying campaign was started. Median analytic follow-up time was 55 months in Jinja, 57 months in Kanungu, and 39 months in Tororo. More information on this cohort study has been published in Kamya et al. (13).

We collected outcomes data via both active and passive surveillance. All participants visited the clinic quarterly for collection of thick blood smears and dried blood spot samples. We asked caregivers to seek care for their children at the study clinic for any illness, free of charge. Children who were febrile (>38.0°C) at the sick visit, or who reported fever in the previous 24 h, had thick blood smears obtained for microscopy. Malaria episodes, defined as parasitemia accompanied by self-reported or measured fever, were treated with artemether-lumefantrine per local guidelines if uncomplicated. Complicated or recurrent malaria (occurring within 14 days of last therapy) was treated with quinine (14). Loop-mediated isothermal amplification (LAMP), a more sensitive measure of parasitemia, was performed retrospectively on dried blood spot samples from routine visits at which microscopy was negative. LAMP was performed on samples obtained from children throughout the study, but from adults for only a portion of the follow-up time.

Entomologic data was collected for each household monthly. We collected mosquitoes using miniature CDC light traps and tested them for sporozoites by ELISA (15). For each household, an annual entomological inoculation rate (EIR) was calculated as the product of the yearly household human biting rate (geometric mean of female Anopheles mosquitoes caught in a household per day) and the site sporozoite rate (average proportion of mosquitos positive for *P. falciparum* at each site) (14).

### HLA Genotyping

HLA genotyping was performed using a targeted next generation sequencing method. Briefly, locus-specific primers were used to amplify a total of 23 polymorphic exons of HLA-A, B, C (exons 1–4), DPA1 (exon 2), DPB1 (exons 2, 3), DQA1 (exon 2), DQB1 (exons 2, 3), DRB1 (exons 2, 3), and DRB3, 4, 5 (exon 2) genes with Fluidigm Access Array (Fluidigm Singapore PTE Ltd, Singapore). The 23 Fluidigm PCR amplicons were pooled and subjected to sequencing on an illumina MiSeq sequencer (Illumina, San Diego, CA). HLA alleles and genotypes were called using the Omixon HLA Explore (beta version) software (Omixon, Budapest, Hungary).

### Statistical Analysis

We analyzed the association of all HLA class I (HLA-A, -B, -C) and Class II (HLA-DPA1, -DPB1, -DQA1, -DQB1, -DRB1) alleles with a frequency of >3.0% in our sample with five different outcomes related to malaria immunity:

1. Parasite prevalence: Having at least one symptomatic or asymptomatic parasitemic (by microscopy) visit per quarter.
2. Annual malaria incidence rate: Number of symptomatic malaria episodes per person-year.
3. Parasite density: Log parasite density measured during parasitemic visits (in analyses, stratified by two visit types: routine quarterly visits and symptomatic malaria visits).
4. Probability of symptoms if infected: Whether a participant was febrile if they were parasitemic. (To count each parasitemic episode only once, we excluded episodes of asymptomatic parasitemia where fever developed within 7 days and repeat episodes of febrile parasitemia within 7 days of initial episode.)
5. Temperature at parasitemic visits: Objective temperature at parasitemic visits, adjusted for parasite density.

We also assessed the relationship of HLA homozygosity (categorized as 3–4, 5 or 6 unique alleles for Class I and 4–7, 8, 9, or 10 unique alleles for Class II) with each of the above outcomes using trend tests.

We estimated multi-level models with random effects at the individual- and household-levels. We used logistic regression for parasite prevalence and probability of symptoms if infected, Poisson models for malaria incidence, and linear models for log parasite density and temperature. All models were adjusted for site, household log entomological inoculation rate, and sex. Controlling for ethnicity (Bantu vs. non-Bantu) based on the language of consent form did not improve model fit as compared to controlling for site due to collinearity. Models included a continuous linear term for age, a binary indicator of age group (child vs. adult), and an interaction between the two to allow for effects to vary between age groups, because the study did not enroll participants between the ages of 11 and 17. Models for temperature also controlled for log parasite density at that visit.

In summary, the models followed this general form (an example model for parasite prevalence shown):

$$\begin{array}{l} logit(Parasite\;prevalence_{ijk})=HLA\;allele_{ij}+Site_{j}+Log(aEIR_{j})\\ \;+\;Sex_{ij}+Age_{ijk}+Child_{ij}+Age*Child_{ijk}+u_{i}+y_{j} \end{array}$$

where j indicates households, i indicates individuals, and k indicates specific visits. For example, age_ijk_ denotes the age of child i from household j during visit k. As aEIR_j_ represents the average annual EIR recorded for household j (time-invariant), we assume relatively stable transmission intensity over the course of study follow-up.

We generated empirical *p*-values using Monte Carlo permutation tests. We permuted exposure variables 10,000 times and calculated the two-sided *p*-value as the number of permutations that yielded coefficients greater than or equal to the absolute value of the observed coefficient divided by the number of permutations (excluding those that didn't converge, <1%) using the Stata ritest package version 1.1.4 (16). If the calculated *p*-value was equal to 0, we made the conservative assumption that it was one divided by the number of permutations. We controlled for multiple testing for all alleles separately for each outcome using the false discovery rate (FDR) approach with the Stata qqvalue package (17). All analysis was done in Stata 15.1 (18).

## Results

### Clinical Cohort

We analyzed data from a cohort of 892 individuals (659 children and 233 adults) residing in 292 households followed longitudinally at three study sites in Uganda with varying malaria transmission intensity (Table 1). Blood smears were performed every 3 months to assess *P. falciparum* parasitemia and participants came to clinic when ill or febrile to be assessed for malaria. As expected based on the differing transmission intensities, the prevalence of parasitemia was highest among children in Tororo (64%), followed by Kanungu (36%), then Jinja (13%). The incidence of malaria (parasitemia accompanied by fever) followed the same pattern. Adults had lower parasite prevalence and malaria incidence than children.

**Table 1:** Characteristics of study population.

	**Jinja**	**Kanungu**	**Tororo**
**Households (*****N*****)**	**97**	**100**	**95**
Median household EIR (range)	2.1 (1.5–22.0)	6.3 (3.0–44.5)	67.6 (11.5–588.4)
**Adults (*****N*****)**	**89**	**78**	**66**
Median incidence of symptomatic malaria episodes per person-year (range)	0.0 (0.0–2.0)	0.2 (0.0–3.7)	0.0 (0.0–3.0)
Parasite prevalence (% quarters parasite+)	4.9%	12.8%	12.0%
**Children (*****N*****)**	**188**	**268**	**203**
Median age at enrollment, years (range)	4.4 (0.5–10.0)	4.7 (0.5–9.9)	5.1 (0.6–10.0)
Median incidence of symptomatic malaria episodes per person-year (range)	0.2 (0.0–5.1)	1.2 (0.0–8.1)	2.1 (0.0–8.8)
Parasite prevalence (% quarters parasite + range)	12.7%	36.4%	64.2%
Median follow up time, months (range)^*^	55.3 (2.3–58.6)	56.6 (0.0–58.3)	39.0 (1.8–40.3)
Bantu ethnicity, *n* (%)	181 (65.6%)	342 (98.8%)	2 (0.74%)
Sickle cell, *n* (%)			
AA	201 (72.6%)	324 (93.6%)	195 (72.5%)
AS	73 (26.4%)	22 (6.4%)	70 (26.0%)
SS	0 (0.0%)	0 (0.0%)	2 (0.7%)
No result	3 (1.1%)	0 (0.0%)	2 (0.7%)

* *Three people in the sample had only one visit; Tororo visits after December 31, 2014 were excluded because transmission changed dramatically at this site after intensive indoor residual spraying campaigns were started*.

HLA genotyping was performed using a targeted next generation sequencing method. Frequencies of HLA alleles are presented in Supplementary Table 1. To investigate whether any HLA alleles influence the risk of *P. falciparum* infection or its clinical manifestations, we tested associations of all alleles present in >3.0% of our study cohort with five malaria outcomes: parasite prevalence (at least one parasite positive visit per quarter by microscopy), malaria incidence rate, parasite density (stratified by routine quarterly visits and malaria visits), probability of symptoms if infected, and temperature at parasitemic visits (conditional on parasite density). Associations were tested using multilevel models (*p*-values denoted as *p*). We then used Monte Carlo permutation tests to calculate empirical *p*-values (p^*^) and controlled for multiple testing stratified by outcome by applying the false discovery rate approach to the empirical *p*-values, yielding *q*-values (*q*).

### Class I HLA Alleles B^*^53:01 and C^*^06:02 Are Associated With Increased Parasite Prevalence

Of the 47 HLA class I alleles we assessed, two alleles (B^*^53:01 and C^*^06:02) were associated with an increased prevalence of *P. falciparum* parasitemia following stringent correction for multiple comparisons. Individuals with HLA-B^*^53:01 (prevalence = 15%) had 1.59 times the odds of parasitemia compared to those without (Table 2; *p* < 0.01, p^*^ < 0.01, *q* = 0.01). Interestingly, HLA-B^*^53:01-positive individuals also had a *lower* probability of symptoms if they were parasitemic (Supplementary Table 2a; OR = 0.67, *p* = 0.03, p^*^ = 0.03, *q* = 0.29), although this association was not significant after correction for multiple comparisons. Notably, this allele has previously been associated with protection against severe malaria in Gambian individuals (8).

**Table 2:** Association of class I HLA alleles and parasite prevalence.

**HLA Allele**	**Odds ratio**	***P*-value**	**Empirical *p*-value**	**Empirical FDR *q*-value**	***N***
A^*^0101	1.08	0.67	0.65	0.86	892
A^*^0201	0.87	0.19	0.15	0.57	892
A^*^0202	1.25	0.12	0.09	0.43	892
A^*^0205	0.97	0.92	0.90	0.97	892
A^*^0301	0.90	0.48	0.43	0.79	892
A^*^2301	1.05	0.70	0.67	0.86	892
A^*^2901	0.88	0.63	0.57	0.85	892
A^*^2902	0.88	0.36	0.30	0.69	892
A^*^3001	0.96	0.77	0.74	0.90	892
A^*^3002	0.89	0.39	0.35	0.73	892
A^*^3004	0.93	0.80	0.76	0.91	892
A^*^3201	1.33	0.28	0.23	0.59	892
A^*^3303	1.04	0.89	0.87	0.97	892
A^*^3402	1.55	**0.02**	**0.01**	0.23	892
A^*^3601	1.37	0.07	0.06	0.32	892
A^*^6601	1.24	0.20	0.15	0.57	892
A^*^6802	0.98	0.85	0.84	0.95	892
A^*^7401	0.78	0.10	0.05	0.32	891
B^*^0702	0.78	0.18	0.14	0.57	892
B^*^0801	0.99	0.97	0.96	0.97	892
B^*^1402	0.92	0.64	0.61	0.86	892
B^*^1503	0.86	0.29	0.23	0.59	892
B^*^1510	1.24	0.25	0.21	0.59	892
B^*^1801	0.99	0.97	0.97	0.97	892
B^*^3501	1.06	0.77	0.74	0.90	892
B^*^4101	0.83	0.51	0.42	0.79	892
B^*^4201	0.99	0.97	0.96	0.97	892
B^*^4403	0.98	0.93	0.92	0.97	892
B^*^4501	0.82	0.17	0.14	0.57	892
B^*^4901	0.74	0.07	0.05	0.32	892
B^*^5101	0.78	0.29	0.18	0.59	892
B^*^5301	1.59	**<0.01**	**<0.01**	**0.01**	892
B^*^5703	1.09	0.65	0.63	0.86	892
B^*^5801	0.87	0.33	0.26	0.64	892
B^*^5802	1.28	**0.04**	**0.02**	0.31	892
B^*^8101	1.29	0.07	**0.04**	0.32	892
C^*^0210	0.91	0.48	0.42	0.79	891
C^*^0302	1.06	0.75	0.72	0.90	891
C^*^0304	1.11	0.53	0.50	0.84	891
C^*^0401	1.25	**0.03**	**0.01**	0.23	891
C^*^0602	1.37	**<0.01**	**<0.01**	**0.02**	892
C^*^0701	0.82	0.06	**0.04**	0.32	891
C^*^0702	0.83	0.28	0.23	0.59	891
C^*^0704	0.93	0.64	0.60	0.86	891
C^*^0802	0.83	0.25	0.20	0.59	891
C^*^1601	0.86	0.27	0.23	0.59	891
C^*^1701	1.08	0.51	0.49	0.83	887

*Sample size varies due to failed amplification or lack of DNA. Values < 0.05 are displayed in bold*.

In addition, individuals positive for HLA-C^*^06:02 (prevalence = 29%) had 1.37 times higher odds of parasitemia compared to those without this allele (Table 2; *p* < 0.01, p^*^ < 0.01, *q* = 0.02). HLA-C^*^06:02-positive individuals also had a higher incidence of symptomatic malaria, although this association was not significant after correction for multiple comparisons [Table 3; incidence rate ratio (IRR) = 1.21, *p* = 0.02, p^*^ = 0.01, *q* = 0.33]. To our knowledge, no association of malaria with HLA-C^*^06:02, nor any other HLA-C allele, has been previously reported in the literature. HLA-C^*^06:02 is a member of the HLA-C2 KIR ligand group recognized by the strongly inhibitory receptor KIR2DL1. We have recently shown that the HLA-C2 ligand group is associated with a higher prevalence of *P. falciparum* parasitemia (19); hence the association of HLA-C^*^06:02 with increased parasitemia could result from the provision of inhibitory signals to effector cells via KIR2DL1, rather than its role as an antigen-presenting molecule. We were not able to directly compare malaria outcomes in HLA-C^*^06:02+ individuals with and without KIR2DL1, however, as KIR2DL1 was present in 99% of individuals in our cohort.

**Table 3:** Association of class I HLA alleles and incident malaria.

**HLA Allele**	**Incidence rate ratio**	***P*-value**	**Empirical *p*-value**	**Empirical FDR *q*-value**	***N***
A^*^0101	1.03	0.80	0.79	0.90	889
A^*^0201	0.80	**0.01**	** <0.01**	0.33	889
A^*^0202	1.14	0.24	0.20	0.54	889
A^*^0205	1.22	0.29	0.22	0.57	889
A^*^0301	0.89	0.30	0.26	0.63	889
A^*^2301	1.08	0.45	0.39	0.74	889
A^*^2901	0.95	0.80	0.77	0.90	889
A^*^2902	1.05	0.64	0.61	0.84	889
A^*^3001	0.83	0.12	0.07	0.47	889
A^*^3002	1.02	0.87	0.86	0.93	889
A^*^3004	0.73	0.21	0.09	0.47	889
A^*^3201	1.37	0.12	0.10	0.47	889
A^*^3303	1.19	0.38	0.29	0.64	889
A^*^3402	1.44	**0.01**	**0.01**	0.33	889
A^*^3601	1.07	0.60	0.58	0.83	889
A^*^6601	1.18	0.22	0.16	0.54	889
A^*^6802	0.95	0.63	0.60	0.83	889
A^*^7401	0.84	0.14	0.07	0.47	888
B^*^0702	0.79	0.12	0.07	0.47	889
B^*^0801	0.94	0.67	0.63	0.86	889
B^*^1402	1.02	0.92	0.91	0.94	889
B^*^1503	0.91	0.40	0.32	0.67	889
B^*^1510	1.22	0.18	0.16	0.54	889
B^*^1801	1.08	0.51	0.48	0.80	889
B^*^3501	0.98	0.91	0.89	0.93	889
B^*^4101	0.87	0.55	0.42	0.77	889
B^*^4201	0.96	0.73	0.71	0.90	889
B^*^4403	1.09	0.60	0.57	0.83	889
B^*^4501	0.86	0.19	0.16	0.54	889
B^*^4901	0.79	0.07	0.05	0.47	889
B^*^5101	0.78	0.21	0.10	0.47	889
B^*^5301	1.13	0.21	0.18	0.54	889
B^*^5703	0.96	0.80	0.79	0.90	889
B^*^5801	1.03	0.81	0.78	0.90	889
B^*^5802	1.15	0.14	0.10	0.47	889
B^*^8101	1.23	0.06	**0.03**	0.47	889
C^*^0210	0.95	0.65	0.60	0.83	888
C^*^0302	1.05	0.73	0.68	0.89	888
C^*^0304	1.18	0.20	0.18	0.54	888
C^*^0401	1.02	0.80	0.77	0.90	888
C^*^0602	1.21	**0.02**	**0.01**	0.33	889
C^*^0701	0.84	**0.04**	**0.02**	0.45	888
C^*^0702	0.80	0.12	0.07	0.47	888
C^*^0704	1.05	0.70	0.69	0.89	888
C^*^0802	1.01	0.94	0.93	0.94	888
C^*^1601	0.89	0.28	0.23	0.59	888
C^*^1701	1.02	0.82	0.81	0.91	884

*Sample size varies due to failed amplification or lack of DNA. Values < 0.05 are displayed in bold*.

Additional class I alleles were associated with higher risk of parasitemia (Table 2; A^*^34:02, B^*^58:02, B^*^81:01, and C^*^04:01) or malaria incidence (Table 3; A^*^34:02 and B^*^81:01), but these associations were no longer significant following multiple comparisons correction.

### No Class I HLA Alleles Are Associated With Protection From Parasitemia or Malaria

Notably, we found no alleles that were associated with *protection* from *P. falciparum* malaria or parasitemia according to any of our outcome measures after correction for multiple comparisons (Tables 2, 3, Supplementary Tables 2a–d). HLA-C^*^07:01 (prevalence = 26%) was associated with lower parasite prevalence (Table 2; OR = 0.82, *p* = 0.06, p^*^ = 0.04, *q* = 0.32) and lower incidence of malaria (Table 3; IRR = 0.84, *p* = 0.04, p^*^ = 0.02, *q* = 0.45) before correction. In addition, HLA-A^*^02:01 (prevalence = 23%), an allele previously linked to increased risk of severe malarial anemia (11), was associated with a lower incidence of malaria (Table 3; IRR = 0.80, *p* = 0.01, p^*^ < 0.01, *q* = 0.33) and a lower probability of symptoms if infected (Supplementaru Table 2a; OR = 0.68, *p* = 0.03, p^*^ = 0.02, *q* = 0.29) prior to correction. To investigate whether presentation of a broad array of class I epitopes facilitates protection, we assessed whether class I HLA homozygosity influenced any of our outcome measures by comparing indviduals with 3–4, 5, or 6 unique alleles. No significant heterozygote advantage was observed.

### No Association of HLA Class II Alleles With Parasitemia or Clinical Malaria

We performed a parallel analysis of associations between HLA class II alleles (HLA-DP, -DQ, -DR) and the malaria and parasitemia outcomes outlined above. Forty-nine alleles were present in >3% of the study population and were included in our analyses. We found no association between class II HLA homozygosity and any of our outcome measures, nor was any individual class II allele associated with any outcome after adjustment for correction for multiple comparisons. Two alleles, DQA1^*^04:01 and DRB1^*^03:02, were associated with higher parasite prevalence before adjustment for multiple comparisons (Table 4). None were associated with increased incidence of malaria (Table 5). Individuals with HLA-DRB1^*^10:01 (prevalence 5%) had a lower prevalence of parasitemia compared with those without (Table 4; OR = 0.64, *p* = 0.03, p^*^ = 0.02, *q* = 0.28), and this allele has been associated with lower parasite density in a prior study (7). However, this association was no longer significant following adjustment for multiple comparisons. DPB1^*^03:01, DQA1^*^01:05, and DRB1^*^11:02 were associated with less incident malaria before adjustment for multiple comparisons (Table 5).

**Table 4:** Association of class II HLA alleles and parasite prevalence.

**HLA Allele**	**Odds ratio**	***P*-value**	**Empirical *p*-value**	**Empirical FDR *q*-value**	***N***
DPA1^*^0103	0.99	0.94	0.94	0.97	892
DPA1^*^0201	0.96	0.67	0.65	0.86	892
DPA1^*^0202	1.03	0.75	0.73	0.90	892
DPA1^*^0301	1.01	0.94	0.93	0.97	892
DPA1^*^0401	0.83	0.40	0.31	0.69	892
DPB1^*^0101	0.97	0.76	0.74	0.90	892
DPB1^*^0201	0.99	0.91	0.91	0.97	892
DPB1^*^0301	0.77	0.10	0.05	0.32	892
DPB1^*^0401	1.04	0.80	0.79	0.92	892
DPB1^*^10501	0.98	0.84	0.82	0.94	892
DPB1^*^1101	0.94	0.82	0.78	0.92	892
DPB1^*^1301	1.13	0.49	0.44	0.79	892
DPB1^*^1701	0.81	0.21	0.16	0.57	892
DPB1^*^1801	1.08	0.61	0.57	0.85	892
DPB1^*^3001	1.15	0.57	0.53	0.84	892
DQA1^*^0101	1.06	0.66	0.60	0.86	890
DQA1^*^0102	1.00	0.97	0.97	0.97	890
DQA1^*^0103	1.26	0.23	0.20	0.59	890
DQA1^*^0105	0.81	0.10	0.07	0.35	890
DQA1^*^0201	0.91	0.47	0.42	0.79	892
DQA1^*^0303	0.91	0.55	0.49	0.83	891
DQA1^*^0401	1.35	**0.01**	**0.01**	0.22	891
DQA1^*^0501	1.08	0.59	0.57	0.85	886
DQA1^*^0505	1.07	0.56	0.51	0.84	886
DQB1^*^0201	1.10	0.52	0.48	0.83	892
DQB1^*^0202	0.98	0.83	0.81	0.93	892
DQB1^*^0301	1.07	0.57	0.52	0.84	892
DQB1^*^0302	0.90	0.70	0.66	0.86	892
DQB1^*^0319	1.13	0.36	0.32	0.69	892
DQB1^*^0402	1.24	0.11	0.07	0.35	892
DQB1^*^0501	0.92	0.40	0.34	0.73	892
DQB1^*^0602	1.12	0.24	0.20	0.59	892
DQB1^*^0603	0.88	0.59	0.57	0.85	892
DQB1^*^0604	1.01	0.96	0.96	0.97	892
DQB1^*^0609	0.77	0.07	0.05	0.32	892
DRB1^*^0102	0.89	0.42	0.36	0.74	892
DRB1^*^0301	1.07	0.64	0.61	0.86	892
DRB1^*^0302	1.33	**0.04**	**0.03**	0.31	892
DRB1^*^0701	0.79	0.08	0.05	0.32	892
DRB1^*^0804	1.20	0.28	0.23	0.59	892
DRB1^*^0901	1.13	0.60	0.52	0.84	892
DRB1^*^1001	0.64	**0.03**	**0.02**	0.28	892
DRB1^*^1101	1.20	0.10	0.05	0.32	892
DRB1^*^1102	0.84	0.20	0.14	0.57	892
DRB1^*^1201	0.89	0.45	0.41	0.79	892
DRB1^*^1301	1.16	0.34	0.31	0.69	892
DRB1^*^1302	0.89	0.27	0.21	0.59	892
DRB1^*^1303	1.26	0.18	0.16	0.57	892
DRB1^*^1503	1.10	0.35	0.30	0.69	892

*Sample size varies due to failed amplification or lack of DNA. Values < 0.05 are displayed in bold*.

**Table 5:** Association of class II HLA alleles and incident malaria.

**HLA Allele**	**Incidence rate ratio**	***P*-value**	**Empirical *p*-value**	**Empirical FDR q-value**	***N***
DPA1^*^0103	0.99	0.88	0.87	0.93	889
DPA1^*^0201	0.95	0.52	0.48	0.80	889
DPA1^*^0202	1.00	0.95	0.95	0.95	889
DPA1^*^0301	0.96	0.59	0.54	0.83	889
DPA1^*^0401	0.99	0.94	0.93	0.94	889
DPB1^*^0101	1.02	0.74	0.72	0.90	889
DPB1^*^0201	1.05	0.57	0.54	0.83	889
DPB1^*^0301	0.80	0.09	**0.04**	0.47	889
DPB1^*^0401	1.08	0.48	0.45	0.80	889
DPB1^*^10501	0.95	0.54	0.48	0.80	889
DPB1^*^1101	1.03	0.88	0.87	0.93	889
DPB1^*^1301	1.13	0.38	0.32	0.67	889
DPB1^*^1701	0.85	0.23	0.16	0.54	889
DPB1^*^1801	0.97	0.78	0.76	0.90	889
DPB1^*^3001	0.95	0.80	0.78	0.90	889
DQA1^*^0101	1.05	0.66	0.60	0.83	887
DQA1^*^0102	1.05	0.49	0.43	0.77	887
DQA1^*^0103	1.30	0.08	0.07	0.47	887
DQA1^*^0105	0.83	0.07	**0.04**	0.47	887
DQA1^*^0201	1.01	0.89	0.88	0.93	889
DQA1^*^0303	1.07	0.58	0.53	0.83	888
DQA1^*^0401	1.10	0.32	0.28	0.63	888
DQA1^*^0501	1.16	0.20	0.16	0.54	883
DQA1^*^0505	0.92	0.34	0.27	0.63	883
DQB1^*^0201	1.19	0.13	0.10	0.47	889
DQB1^*^0202	1.06	0.50	0.47	0.80	889
DQB1^*^0301	0.93	0.42	0.37	0.72	889
DQB1^*^0302	0.78	0.29	0.20	0.54	889
DQB1^*^0319	0.96	0.72	0.69	0.89	889
DQB1^*^0402	1.10	0.37	0.33	0.67	889
DQB1^*^0501	0.94	0.40	0.34	0.67	889
DQB1^*^0602	1.10	0.20	0.16	0.54	889
DQB1^*^0603	1.03	0.86	0.85	0.93	889
DQB1^*^0604	1.06	0.58	0.54	0.83	889
DQB1^*^0609	0.85	0.13	0.10	0.47	889
DRB1^*^0102	0.91	0.42	0.33	0.67	889
DRB1^*^0301	1.16	0.20	0.17	0.54	889
DRB1^*^0302	1.11	0.30	0.27	0.63	889
DRB1^*^0701	0.95	0.60	0.57	0.83	889
DRB1^*^0804	1.04	0.75	0.73	0.90	889
DRB1^*^0901	1.21	0.31	0.22	0.57	889
DRB1^*^1001	0.79	0.18	0.12	0.51	889
DRB1^*^1101	1.01	0.88	0.85	0.93	889
DRB1^*^1102	0.82	0.07	**0.03**	0.47	889
DRB1^*^1201	0.85	0.19	0.15	0.54	889
DRB1^*^1301	1.26	0.06	0.05	0.47	889
DRB1^*^1302	0.94	0.43	0.39	0.74	889
DRB1^*^1303	1.07	0.60	0.60	0.83	889
DRB1^*^1503	1.10	0.22	0.19	0.54	889

*Sample size varies due to failed amplification or lack of DNA. Values < 0.05 are displayed in bold*.

### Submicroscopic Parasitemia

As a secondary analysis, we examined the prevalence of parasitemia when the definition was expanded to include submicroscopic infections (i.e., low-density infections that are blood smear-negative but detectable by LAMP, or loop isothermal amplification). Data for this analysis were restricted to the portion of the follow-up time where LAMP data was available for both children and adults (median follow-up time in months: Jinja = 25.0, Kanungu = 43.5, Tororo = 39.0), as LAMP assays were not performed on all adult samples (Supplementary Table 3). In this analysis, HLA-B^*^53:01 remained statistically significant (OR = 1.51, *p* < 0.01, p^*^ < 0.01, *q* = 0.03), but HLA-C^*^06:02 did not (OR = 1.26, *p* = 0.05, p^*^ = 0.02, *q* = 0.18) even though the odds ratio was qualitatively similar. Loss of significance may be related to the reduced power due to fewer person-months of observation. HLA-A^*^34:02 was also associated with increased parasite prevalence detectable by LAMP (OR = 1.94, *p* < 0.01, p^*^ < 0.01, *q* = 0.02), although it was not in the primary analysis (based on microscopy alone) after multiple comparisons correction (Table 2; OR = 1.55, *p* = 0.02, p^*^ = 0.01, *q* = 0.23).

### Sensitivity Analyses

To evaluate the robustness of the observed associations with *P. falciparum* parasite prevalence, we performed several sensitivity analyses. We executed the following models: (1) stratified by site (Supplementary Tables 4a–c) to determine whether our results were robust across transmission intensities and ethnicities, (2) restricted to children, as they suffer the largest burden of parasitemia (Supplementary Table 5), and (3) excluding individuals with hemoglobin S (sickle cell) mutations to ensure our results were not confounded by sickle cell variants (Supplementary Table 6). For all, similar odds ratios were detected for the statistically significant results stated above.

## Discussion

Using data from a large cohort of Ugandan adults and children followed longitudinally with careful prospective surveillance for both *P. falciparum* parasitemia and clinical malaria, we identified two class I HLA alleles, HLA-C^*^06:02 and HLA-B^*^53:01, that were associated with an increased prevalence of *P. falciparum* parasitemia. While the HLA-C^*^06:02 association is novel, the association of HLA-B^*^53:01 with a higher risk of parasitemia is notable in light of prior evidence linking this allele to protection from severe malaria (8). We were unable to replicate other class I and class II HLA associations with malaria that have been previously reported (6, 8–11), in concordance with results from a recent large GWAS study (20). Of note, we found no alleles associated with *protection* from malaria after stringent correction for multiple comparisons.

To our knowledge, the association between HLA-C^*^06:02 and increased prevalence of parasitemia is the first reported association between an HLA-C allele and any malaria outcome. HLA-C is expressed on the cell surface at much lower levels than either HLA-A or HLA-B (21), and it has been hypothesized that HLA-C evolved to act as a ligand for KIR, rather than to exclusively present antigen (22–24). Therefore, in addition to its role in antigen presentation, it is possible that the observed association of HLA-C^*^06:02 with increased parasitemia could result from provision of inhibitory signals to effector cells via KIR which could dampen the cellular immune responses that contribute to parasite restriction. HLA-C^*^06:02 is a member of the HLA-C2 KIR ligand group which exerts strong inhibitory signals through KIR2DL1. HLA-C^*^04:01, the other highly prevalent HLA-C2 ligand group allele in our cohort, was also associated with higher odds of parasitemia prior to correction, although this may be due to its linkage disequilibrium with B^*^53:01 (25). Conversely, HLA-C^*^07:01, a member of the HLA-C1 ligand group which mediates a weaker inhibitory signal via KIR2DL2/3, was associated with lower odds of parasitemia. These associations are all consistent with our recent finding that the HLA-C2 ligand group is associated with increased parasite prevalence, while the HLA-C1 ligand group is associated with lower parasite prevalence (19). The interaction of HLA-C^*^06:02 with the strongly inhibitory receptor KIR2DL1 likely influences NK cell education even in the absence of infection, leading to more potent NK cell function upon activation, although it is not evident how this would result in an increased risk of parasitemia. Finally, it is notable that HLA-C^*^06:02 is one of the most highly expressed HLA-C alleles, resulting in part from its association with the HLA-C promoter region variant rs2395471, as well as sequence variation within its peptide binding groove that facilitates peptide loading (26–28). Its relatively high surface expression may enable HLA-C^*^06:02 to present antigen more efficiently to CD8 T-cells, and also to provide stronger inhibition of NK cells via KIR, either of which could underlie the association of HLA-C^*^06:02 with infectious outcomes and with inflammatory diseases such as psoriasis (29). In support of this, we recently observed that higher HLA-C surface expression was associated with increased *P. falciparum* parasite prevalence (19).

The relationship between HLA-B^*^53:01 and malaria has been the focus of several prior studies (30–33), beginning with a widely cited case-control study conducted in the Gambia that found HLA-B^*^53 to be strongly protective against severe malaria (8). This protective effect was hypothesized to result from presentation of immunodominant liver stage epitopes, LSA-1 (30) and LSA-3 (31). However, the protective association against severe malaria has not been replicated in subsequent case-control studies (6, 9, 20, 34) and could not be assessed in our cohort as severe malaria was extremely rare (13). Instead, we found that HLA-B^*^53:01-positive individuals had a higher odds of parasitemia, but trended toward a lower probability of symptoms when parasitemic. While the reduced risk of malaria symptoms upon infection could be consistent with the prior report that HLA-B53 protects against severe malaria, our findings argue that this protection is not mediated by a sterilizing pre-erythrocytic immune response, and it is difficult to explain on the basis of HLA-B53-restricted CD8 T cells. A potential alternate explanation is that it acts via immune inhibition mediated by KIR or other inhibitory class I HLA receptors; HLA-B53 is a member of the HLA-Bw4 ligand group which delivers inhibitory signals via KIR3DL1. We have recently shown that KIR3DL1 inhibitory ligand HLA-Bw4 increases the risk of *P. falciparum* parasitemia (19). KIR-mediated inhibition may dampen protection from parasitemia while also dampening the inflammatory cascade responsible for symptomatic and severe malaria. Intriguingly, a recent analysis of pooled HLA-A, -B, and -DRB1 data from six randomized trials of RTS,S, the leading malaria vaccine candidate, found that HLA-B^*^53 was associated with a lack of protection in vaccine recipients, as well as lower post-vaccination titers of anti-CSP antibodies (33). Hence, the mechanisms by which HLA-B^*^53 mediates a higher prevalence of parasitemia following natural infection may also be relevant to vaccine-mediated protection. It is notable that HLA-B^*^53 is strongly associated with accelerated progression to AIDS following HIV infection (35, 36), which suggests that a potential common immunopathogenetic mechanism independent of any pathogen-derived epitope may be responsible for our findings.

Our study had several limitations. Although the sample size was much larger than that of most prior malaria HLA association studies, our statistical power to detect an effect was limited for alleles that were of low frequency in the study population. For this reason, we restricted our analysis to alleles with a population frequency >3%. Our sampling framework did not allow us to discriminate whether the higher parasite prevalence observed among HLA-C^*^06:02 and HLA-B^*^53:01 positive individuals resulted from a higher rate of incident infection or if it instead resulted from delayed clearance of parasites from the blood. Additionally, while it would have been interesting to explore the influence of HLA-KIR ligand pairs (i.e. KIR2DL1/HLA-C^*^06:02 and KIR3DL1/HLA-B^*^53:01) on malaria outcomes, the prevalence of both of these inhibitory KIR in our cohort was >98% (19). Given this, we could not estimate these effects. [[[Further, we did not have KIR allele level data to look for more specific associations with HLA alleles.]]] An additional limitation of our study is that linkage disequilibrium poses an inherent challenge to inferring a causal relationship between HLA alleles and malaria outcomes. We cannot exclude the possibility that a nearby locus could be responsible for a particular observed effect. Finally, while population stratification is potentially a concern, controlling for ethnicity in our study did not improve model fit beyond controlling for site alone. A key strength of our study, beyond the large cohort size, was that our longitudinal outcome measures enabled us to separately evaluate HLA associations with parasitemia and its clinical manifestations, while adjusting for variability in exposure using household-level entomological data. In addition, we strictly corrected for multiplicity of testing using the false discovery method, which was not done in many prior studies.

To date, immunogenetic correlates of malaria susceptibility have been elusive. Our findings support a role for class I HLA molecules in influencing the establishment or clearance of parasitemia, and strengthen the evidence that cellular immunity is an important component of a protective response to *P. falciparum*. The association of HLA-C^*^06:02 and HLA-B^*^53:01 with a higher risk of parasitemia could reflect greater KIR-mediated inhibition leading to dampened cellular immunity at pre-erythrocytic stages or an attenuated clearance of parasites via antibody dependent cellular cytotoxicity. Our results in conjunction with previous data present a paradox regarding the role of HLA-B^*^53 in susceptibility to parasitemia and protection from severe malaria. Together, our findings underscore the need to examine correlates of protection for endpoints other than severe malaria. Finally, these results highlight the need for further study of the role of HLA in restricting pre-erythrocytic stages of the *P. falciparum* life cycle, as well as the need for better tools, such as tetramers, to enable mechanistic studies of the T cell response to *P. falciparum* at the epitope level.

## Data Availability Statement

Data and code for this analysis are available at: https://github.com/feeneylab/HLA_2021_FrontiersImmunology

## Ethics Statement

Ethical approval for this study was granted by Makerere University School of Medicine Research and Ethics Committee, London School of Hygiene and Tropical Medicine Ethics Committee, University of California, San Francisco, Committee on Human Research and Uganda National Council for Science and Technology. All participants and/or their parents/guardians gave written informed consent for their household upon enrollment.

## Author Contributions

JD, PC, IR-B, and MF designed experimental questions. IR-B and JD created statistical analysis plan. JD performed all statistical analyses. JD, PC, and MF prepared original manuscript. GD, MK, EA, and JR generated the clinical data set. MM and MC performed KIR and HLA genotyping. GN and MV validated statistical analyses. All authors contributed to the article and approved the submitted version.

## Conflict of Interest

The authors declare that the research was conducted in the absence of any commercial or financial relationships that could be construed as a potential conflict of interest.
